# Wages, Hours, and Prices

**Published:** 1920-01-31

**Authors:** 


**THE PUBLIC WELL-BEING—(*continued*).**

### WAGES, HOURS, AND PRICES.

HIGH wages, shorter hours, and higher prices are the main features of the annual review of 1919, published in the *Labour Gazette*. Decreases in hours have affected 6,400,000 persons, with an aggregate reduction in weekly hours of 41,461,000. The number of workpeople whose wages went up numbered 5,647,000, with a net increase in weekly wages of £2,111,000. The advance in wages continued during the year, though not so notably as in 1918, and doubtless higher prices are partly due to these circumstances.

There were some remarkable changes in retail prices. They fell substantially at the beginning of the year, but afterwards picked up again, and even surpassed the original high level. At the beginning of the year the average increase, compared with July 1914, in the retail prices of food, on the basis of pre-war standard of consumption, was 130 per cent. In June it was 104, but in January—this present month—it has gone up to 136 per cent. For all the items included in the statistics, comprising rent, clothing, fuel, light, etc., as well as food, the corresponding percentages at the three dates were 120, 105, and 125. There seems little hope of improvement yet, for several of the things included are still mounting up in price. Labour has done better than any other section of the community in securing wages to meet the higher cost of living. In fact, it has done so largely at the expense of that part of the community which can least stand it, and which is squeezed on one side by Labour and on the other side by profiteers. Labour should not be grudged its good fortune. But the profiteer should be roughly handled, otherwise the consequences may be serious in several directions.

